# On the Uncertainty of Diagnosis by Physical Signs in the First Stages of Diseases of the Chest

**Published:** 1852-06

**Authors:** J. W. Freer

**Affiliations:** Demonstrator of Anatomy in Rush Medical College


ART. II.—*On the uncertainty of Diagnosis by Physical Signs in the first stages of Diseases of the Chest.* By J. W. FREER, M.D., Demonstrator of Anatomy in Rush Medical College.

AN eminent medical writer has recently offered the opinion that diseases, like our garments, are subject to the fluctuations of fashion. We may presume, however, that this statement simply refers to those imaginary affections which have their origin in the whimsicalities of the afflicted person.

Since the era of physical diagnosis, these imaginary affections are fashionably located within the thoracic cavity. Most practitioners of medicine must be aware of how large a proportion of individuals coming under their cognizance, are afflicted with this sort of monomania respecting the stability of their thoracic organs. And these apprehensions are not confined to the weaker human vessels—to those whose general appearance would indicate the probability of the real existence of disease; on the contrary, we quite as often find the victimized to be those whose general appearance would indicate most perfect organisms, giving large promise of longevity. This is all quite natural; for the uncertainty of human life under the most promising circumstances, is well calculated to force mankind to a constant anxiety in regard to their bodily conditions. Now this very natural anxiety which is apt to pervade the minds of men, respecting their physical well-being, also impels them to seize with great avidity upon every monitor which promises to enlighten in regard to the actual condition of things within their systems. Again, this solicitude for bodily welfare is the forcible occasion of directing the public mind to what are supposed to be great discoveries in the sciences pertaining to the healing art; and, what is not in the least surprising, they, as a general thing, place more implicit reliance in proclaimed discoveries than do even the discoverers themselves, or their scientific followers,—for the good reason, that their means of judging is more limited.

Before the art of physical diagnosis became in vogue, affections of the chest were held by the public as quite mysterious in their nature. Their apprehensions were founded principally upon external manifestations. While these were plausible, security was inferred; but when this new means of exploring the hidden mysteries of this important region was heralded to the world, professing to be unerring, even to mathematical precision—providing the operator is sufficiently expert—appearances, by a certain class of persons, were no longer relied upon, "for," says the hypochondriac, "why not avail myself of this infallible test, and at least assure myself in regard to this division of my precious body."

Now it may be proven that the foregoing causes operating upon a large proportion of minds, have been the indirect means of prostituting this most beneficent discovery to the purposes of imposition, speculation, and avarice. This valuable means of diagnosis has not only been imposed upon through base motives, but perhaps it has been quite as often, though innocently, injured by those who, though skillful in their manipulations, yet being of large faith and placing too much reliance in its dicta, to the exclusion of rational symptoms, frequently impose upon themselves, and their art at the same time, to the great detriment of all concerned. I would not speak thus confidently, could I not at the present moment call to mind numerous cases which abundantly support the above assertions, and at the same time having no doubt that many other practitioners will confess to the same experience.

Of those of the former class who are in the habit of making this useful acquisition to medical science a means for imposing upon the credulity of people, and at the same time enriching their pockets, we have but little to say. They are a class of persons who, having entered the profession on speculation, have taken the precaution to dispose of their consciences before adopting that important step, therefore a word of reproof to such would excite little else than a smile of derision. But of those who conscientiously impose upon themselves and the public, either from want of skill in the exercise of their art, or in being possessed of too much faith in its capabilities in detecting incipient disease, a few remarks may appropriately be made.

This class of Physicians are, generally speaking, what Tristram Shandy would have denominated "hobbyhorsical" in their natures; therefore being attracted by chance in this or that direction, wherever a hobby may be found, it is at once mounted and ridden to destruction.

We will now attempt to show some of the causes which are oftentimes prone to lead this class of physicians into error of diagnosis in diseases—whether tubercular or otherwise—common to the thoracic viscera. These errors being principally confined to attempts to define disease in its incipient period, rather than to more palpable conditions in which error would be quite inexcusable.

The principal source of fallacy no doubt exists in the impossibility, so to speak, of detecting most diseases of the thoracic organs in their earliest stages, by means of physical exploration alone, —especially of tubercular deposit, of which I shall have most to say, as connected more particularly with the mischievous tendencies of false diagnosis in this region. Yet notwithstanding this uncertainty, this sanguine class of physicians after a time become so absorbed in self-confidence that they imagine themselves competent to detect with unerring precision the least departure from the normal condition, by the aid of sounds elicited by auscultation and percussion, to the exclusion of general and constitutional indications, which at this early period are of infinitely more value than any manual dexterity, however skillfully applied, in order to detect the true nature of the case. The first departure from health in the deposition of tubercle cannot be attended with sufficient variation in auscultatory sounds to enable detection by the most discriminating ear, yet at this very time the grand mischief is already established, and it is at this time alone that medical skill can proffer much hope of aid; but with those who are in the habit of placing too much reliance in their favorite art, this precious time is allowed to pass on unheeded, because of having failed to detect the nice shade of difference existing at this time between healthy and unhealthy respiratory murmur, and percussion happening to give no available signs. Yet there may be general manifestations and symptoms enough, if rightly observed, to lead to satisfactory conclusions.

Again: Errors of an opposite nature are quite as frequently committed and from the same cause, which is, an overweening confidence in the sense of hearing, as connected with auscultation and percussion. And perhaps this class of errors, if possible, are more pernicious in their effects upon the community than the former; it not being quite certain that tubercular affections are positively amenable to treatment even when detected in their initiatory period. How is it that mistakes of this character can be committed? Are not the signs and symptoms of phthisis pulmonalis sufficiently positive? They undoubtedly are when fairly instituted; but, as formerly stated, when a diagnosis is made at too early a period by physical signs alone, we may be easily led astray. According to my own experience in such examinations, we cannot rely upon shades of comparative difference in the corresponding sides of the chest as diagnostic of derangement of any character, whether these differences consist in dullness upon percussion or in respiratory sounds. It is generally understood that the right sub-clavicular region is slightly more resonant than the left; yet we frequently find the reverse upon healthy persons. Marked differences in resonance exist between individual chests which are apparently under the same external conditions as to adipose tissue, muscularity, &c., and both within normal bounds. Why cannot local differences exist in an individual chest and yet be within the same limits? If this may be the case, and I have not the least doubt of its truth, then we cannot be too careful in rendering an unfavorable opinion, especially in the absence of adequate corroboratory evidence.

Other causes of error might be produced which are equally efficient in misleading those who are too confident in their art of physical diagnosis, but the limits of this article will not permit further digression in this direction, and we will conclude by making a few remarks respecting the deleterious effects arising from false diagnosis of pulmonary tubercular disease, not only as regards the public but the profession.

Mankind being so constituted that imagination has largely to do with their physical condition, when the mind is once directed to a given point, either by fashion or otherwise, the first step is taken towards actual ailment. Should this point happen to be the chest, such persons, from the implicit reliance which they have been taught by certain of the profession to place in the certainty of physical diagnosis, immediately resort to some far-famed operator in order to ascertain their doom, and the probable period of their existence. Quite likely this far-famed "chest disease doctor" may belong to the class before-mentioned, who prostitutes his art to venal purposes, and pronounces an unfavorable opinion in the case, in order to have an opportunity to medicate it, and thereby render "material aid" to himself. If, on the contrary, the sufferer happens to fall in with a conscientious hobbyist, the risks then are simply in his physician's false estimates. If the verdict is against him, forever after he is doomed to lead a life of apprehension and inquietude. The monomania is fixed upon him like an incubus, and he goes about from doctor to doctor to be re-examined, but will place no faith in those who will not confirm the first verdict. "Ah!" says he, "you wish to deceive me; you have not the heart to tell me your honest opinion."

This last sentence is a quotation from the lips of a patient which came under my notice a few days since, and will serve as an instance of the misery produced in thus consigning people to the tomb without proper understanding. The case is interesting and I will cite it:

The gentleman had during the past winter been sick, as he informed me, with a typhoid fever, and was attended by one of the most judicious practitioners in our city. Upon recovery from the fever a bronchial affection set in, which was combatted by his physician no doubt with proper remedies. But after a while he became seized with the mania of consumption and was examined by his attending physician, who promptly pronounced his apprehensions groundless. This did not satisfy him; so the patient knowing of another doctor who is said to be much celebrated in chest diseases, immediately sent for him in order to be more than doubly assured that all was right. The latter gentleman, after a careful examination, with rueful face informed him that he was enabled to detect a slight difference in the two sides of the chest; that there was some evidence of a tendency to consumption. Of course he was pronounced honest, for he was not backward in giving his opinion. As is usual with persons suffering under this sort of mania, he was still unsatisfied, believing after a time that his honest doctor was only so in degree, that he withheld from him in regard to the gravity of his case, through consideration for his feelings. Again he passed around from one physician to another, of known celebrity in their profession, but happening not to be "chest doctors" he failed in having the unfavorable judgment of his "honest doctor" confirmed. This patient, to the best of my knowledge, was lastly under my charge. Perceiving his delusion, my labors were principally directed to its eradication, and the most potent and available means seemed to be that of forcibly directing his attention to another point. Opportunely his throat was slightly inflamed, and taking advantage of this circumstance, a solution of nit. arg. was vigorously applied until ultimately his consumption was exchanged for a "fashionable" sore throat, to which he now attributes most of his difficulties.

Numerous cases which have come under my notice, similar to the above, might be cited, where persons have been ignorantly or villainously imposed upon in like manner, and to their serious detriment; but it would be needless to bring them forward as the one quoted is sufficient for an example, at the same time doubting not that a large proportion of the medical profession are sufficiently supplied with cases of a similar character within their own experience, to which they can refer at any time.

From the tenor of this article it may possibly be inferred by some that it has been my aim to speak lightly of the value of the art of auscultation; but should there be such, I would beg leave to inform them that they are laboring under misapprehension, my only aim having been to call attention to the abuses practiced upon this invaluable means of diagnosis.

